# Multinational appraisal of the epidemiological distribution of opioid fatalities: a systematic review and meta-analysis

**DOI:** 10.3389/fpsyt.2023.1290461

**Published:** 2024-01-05

**Authors:** Hope Onohuean, Frasia Oosthuizen

**Affiliations:** ^1^Biopharmaceutics Unit, Department of Pharmacology and Toxicology, Kampala International University Western Campus, Ishaka-Bushenyi, Uganda; ^2^Discipline of Pharmaceutical Sciences, College of Health Sciences, University of KwaZulu-Natal, Durban, South Africa

**Keywords:** appraisal, epidemiological, distribution, opioid-fatal-overdose, meta-analysis

## Abstract

**Background:**

The global or multinational scientific evidence on the distribution of opioid fatality is unknown. Hence, the current study collects epidemiological characteristics to shed light on the ongoing global or multinational opioid crisis and to promote the development of public health prevention/management strategies.

**Method:**

All documents on PRISMA standards were retrieved via electronic databases.

**Results:**

Among the 47 articles relevant to our studies, which depict a total population size of 10,191 individuals, the prevalence of opioid fatal overdose was 15,022 (14.74%). Among the 47 articles, 14 of them reported the gender of the participants, with 22,125 (15.79%) male individuals and 7,235 (5.17%) female individuals, and the age distribution of the participants that was most affected by the overdose was as follows: 29,272 (31.13%) belonged to the 18-34-year-old age group and 25,316 (26.92%) belonged to the less than 18-year-old age group. Eighteen studies qualified for the meta-analysis of the multinational prevalence of fatal opioid overdose, depicting an overall pooled prevalence estimate of 19.66%, with 95% CIs (0.13–0.29), *I*^2^ = 99.76% determined using the random-effects model, and Q statistic of 7198.77 (*p* < 0.0001). The Egger test models of publication bias revealed an insubstantial level of bias (*p* = 0.015). The subgroup analysis of the study design (cohort or other) revealed that others have the highest prevalence estimate of 34.37, 95% CIs (0.1600–0.5901), *I*^2^ = 97.04%, and a sample size of less than 1,000 shows the highest prevalence of 34.66, 95% CIs (0.2039–0.5234), *I*^2^ = 97.82%, compared to that of more than 1,000 with a prevalence of 12.28, 95% CIs (0.0675–0.2131), *I*^2^ = 99.85%. The meta-regression analysis revealed that sample size (less-than or greater-than 1,000), (*p* = 0.0098; R^2^ = 3.83%) is significantly associated with the observed heterogeneity.

**Conclusion:**

Research-based findings of fatal opioid overdose are grossly lacking in middle- and low-income nations. We established that there is a need for opioid fatality surveillance systems in developing nations.

## Introduction

Opioid overdoses and fatalities continue to increase worldwide. Opioid fatalities result from excessive and unopposed stimulation of the opioid receptor signaling pathway in the brain, characterized by difficulty breathing (1, 2). The use of opioids as painkillers significantly increased opium manufacturing (3). There are considerable side effects to using opioid painkillers, including opioid-related overdose (ORO) and associated disorders. The most common causes of opioid overdose are frequently non-medical or illicit, prolonged use, misuse, and use without medical supervision (4). In recent years, common fatal opioid overdose deaths have been mostly linked to fentanyl or synthetic opioid use (5).

Worldwide drug use fatalities are estimated to be approximately 0.5 million, and more than 70% are attributed to opioids, with overdose accounting for more than 30% of those fatalities (6). In 2019, approximately 62 million people used opioids globally, while nearly 36.3 million were affected by its associated disorders (7), and in the United States (US), overdose deaths increased from an estimated prevalence of 70,029 in 2020 to 80,816 in 2021 (8). The trend of opioid mortality is well documented in industrialized countries such as the United States (US)—where it has been a long-term epidemic—Canada, Germany, and United Kingdom (UK) (9–13), while the trend in the middle- and low-income nations is unknown.

Although the opioid crisis has been well documented, the epidemiological characteristics represent only a portion of the global public health issue and are skewed to developed nations. Death statistics only partially illustrate the problem of opioid-related poisoning related to public health. In addition, the features influencing the emergence of drug-related fatalities are poorly understood in developing or underdeveloped countries (14). A significant portion of deaths linked to drug overdose are among adults in their 20s and 30s (15–17).

The existing evidence on opioid fatalities is sparse on epidemiological data from developing nations or on a global scale for future preparedness and prevention of the growing crises. However, the lack of up-to-date global information on specific opioid drugs and contributing to fatal overdoses may hinder the conception of preventative strategies. The need for timely and precise global opioid fatality surveillance data has become more important for stakeholders, policymakers, and researchers who are looking for more efficient responses to the unending pandemic. Therefore, our study aimed to synthesize and evaluate the scientific evidence on the changes in temporal, demographic, and prevalence data of opioid fatalities to appraise the epidemiological characteristics on a global or multinational scale, thereby highlighting the crucial use of evidence data from various public health and safety sources to guide nations on opioid overdose prevention programs and policies to improve surveillance systems among poor resource settings.

## Materials and methods

### Search strategy

Following the standard Preferred Reporting Items for Systematic Reviews and Meta-Analysis (PRISMA) guidelines (18–20), a literature search on the electronic databases, namely, Web of Science (WOS), Scopus, PubMed, and article references, was conducted by employing Boolean keywords that took the form of title words or headings for medical subjects. The search included studies published between 1990 and 23rd May 2022, and the search period was updated to 30th September 2022 at approximately 8.15 GMT + 2.

The Supplementary material S1 contains essential terms such as Epidemiology, Prevalence, Opioids, Fatal, and Overdoses. Title-specific search was employed to appraise various population, survey, prevalence, and epidemiology data on global opioid fatalities in this study. The datasets were merged using the Bibliometrix R package (R program 4.0.5), whereas ScientoPy and fBasics R-packages were used to remove duplicates and normalize variables (19–23). Two independent reviewers (HO and FO) conducted the literature search.

### Study selection criteria

The eligibility guidelines used to include studies were as follows: studies that reported on natural opioids [morphine, codeine, and thebaine (paramorphine)]; semi-synthetic opioids (hydromorphone, hydrocodone, Oxycodone, and heroin); fully synthetic opioids (fentanyl, pethidine, levorphanol, methadone, tramadol, and dextropropoxyphene); opioid fatalities or the main outcomes of interest including prevalence estimates and mortality rates; (or correlates*) of fatal opioid overdose detected by any of the following methods: urine test, emergency log book, police record, toxicological method, social autopsy, postmortem records, and prescription and illicit opioids identified using ICD-10 T-codes; and methadone-caused deaths determined by Substance Abuse and Mental Health Services (SAMHSA). Articles were restricted to English-language only, with no restriction to study method, region, or location.

### Outcomes of interest

The outcome of interest includes the prevalence of estimates of death, mortality rates, and (or correlates*) of fatal opioid overdose.

### Data extraction and outcomes of interest

Two investigators (HO, and FO) independently identified and collated meta-data on the first authors’ names, publication year, total population, number of positive cases (occurrence of fatal overdoses), country of study, studied source, study period, and study type from the qualified articles’ results, discussions, figures, and tables as the meta-analysis performance indicators. Moreover, competencies and discrepancies were evaluated by consensus of the two investigators. Subsequently, homogeneity or consistency and heterogeneity among the populations under study were documented, and further statistical analysis was based on the criteria for the study as envisaged by the investigators.

### Assessment of data quality

The data quality for this meta-analysis was assessed using the Newcastle-Ottawa Scale (NOS) approved by the Agency for Healthcare Research and Quality (AHRQ).^1^ The quality of the studies was graded into three categories—study group selection, group comparability, and outcome measurement—using a star system (24).

### Statistical analysis

The prevalence of global population-based opioid fatal overdose from 18 studies was calculated using the raw proportions, and 95% confidence intervals (CIs) were calculated using the Wilson method. The weighted overall effect size (weighted average proportion) was calculated from the original studies using the random-effects meta-analysis according to the individual effect sizes and associated sample variances by using the argument method = “DL” (using the restricted maximum-likelihood estimator). Since the proportion across studies ranges from 0.005 to 1, the logit transformation was used to obtain the pooled prevalence to improve the statistical characteristics (25). The effects of the study populations’ heterogeneity and homogeneity were measured by meta-regression analyses. The subgroup analyses of epidemiological distribution were performed, and a forest plot was produced. To compare publication bias, funnel plots were created by Egger’s test for asymmetry. The rank correlation test and Kendall’s model were then used to determine the significance of the bias. All analyses were performed using the statistical software R 4.0.5. packages and were two-tailed with *p*-values of 0.05 significance level (26, 27).

## Results

### Literature search summary

#### Summary of included studies

**Figure 1:**
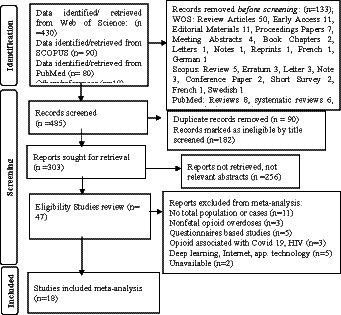
PRISMA flow diagram of the included articles.

Our search results across the three databases spanning 1995–2022 found 600 articles reporting on the global distribution and prevalence estimate of opioid fatal overdose. Upon elimination of duplicates and removal of irrelevant studies, the abstracts and summary of the potentially pertinent studies were reviewed and 47 full articles remained from different countries and regions for data mining. The details of the 47 articles are described in full in the flowchart shown in Figure 1. Thirty-one studies are reported from the USA and three from the UK. Spain and Demark have reported two studies, while all other nations, namely Israel, Switzerland, Australia, Norway, Germany, Ireland, and Colombia, have one each. Table 1 shows the study period and sample size, ranging from 3 months to 9 years and 8 to 2,154,426 per study, respectively.

**Table 1:** Overall characteristics of the studies reviewed and meta-analyzed.

References	Country/Region	Study type	Types of opioids	Study Period	Sample size	Comparability of cohorts	Prevalence/Incident	Method	Score
Walley et al. (28)	USA (Massachusetts)	Retrospective cohort Study	Fentanyl, heroin, tramadol, codeine, hydrocodone, morphine, oxycodone, 6-monoacetylmorphin, hydromorphine, and buprenorphine	2 years	3,710	NA	Death	Toxicological postmortem reports	8
Feingold et al. (12)	Israel	Retrospective cohort Study	Opioids (non-specific)	9 years	875	NA	Death	National database on cause of death	8
Shover et al. (29)	USA	Case report	Isotonitazene	1 January 2020 to 31 July 2020	1,021	NA	Death	Toxicological postmortem reports, autopsy, and relevant medical reports	8
Rodieux et al. (30)	Switzerland	Case study	Tramadol	NA	32	NA	Death	Mortality Registry Record	7
Lowe et al. (31)	Canada (Ontario)	Case study	Hydromorphine and morphine	2007–2012	8	NA	Death	WHO Pharmacovigilance database	8
Dunn et al. (32)	USA (Washington)	Cohort	Hydrocodone, oxycodone, codeine with a combination drug, long-acting morphine, oxycodone CR, tramadol, hydromorphone, methadone, and fentanyl patch	90 days	9,940	NA	NA	Face-to-face interviews	7
Thylstrup et al. (33)	Denmark	Cohort	Heroin, cannabis, methadone, and benzodiazepines	2000–2010	11,199	NA	Death	Consort (consortium to study opioid risks and trends) study	8
Dunn et al. (32)	USA	Cohort	Morphine and hydrocodone	42 Months	51	NA	Death	Patients’ safety review committee of the Office of the Chief Coroner of Ontario	8
Kravitz-Wirtz et al. (34)	Colombia	Cross-sectional	All opioids, including natural and synthetic opioids, and methadone heroin	1 January 2001 to 31 Dec 2017	383,091	NA	NA	NA	7
Roxburgh et al. (35)	Canada (Ontario)	Hospital-based study	Hydromorphone and morphine	5 years	8	NA	NA	Case–control	7
Kimani et al. (36)	USA	Toxicological laboratory	Benzodiazepine (alprazolam, clonazepam, diazepam, estazolam, and midazolam)		169	NA	NA	Postmortem toxicology and prescription drug monitoring record	7
Dayton et al. (37)	USA (Baltimore, Maryland)	Perspective	Naloxone	2017–2019	502	NA	Death	Death certificate, autopsy reports, toxicology, and prescription drug monitoring program data	8
Rintoul et al. (38)	Australia (Victory)	Population-based observational	Oxycodone	2000–2009	100,000	NA	Death, death suicide, cardiorespiratory arrest	Perspective	8
Espelt et al. (39)	Spain	Quasi-experimental	Heroin and methadone	Oct 2008 to March 2009	529	NA	Death	National Vital Statistics System	8
Latkin et al. (40)	USA (Baltimore, Maryland)	Questionnaire	Fentanyl and heroin	6 months	390	NA	Death	NA	7
Glick et al. (41)	USA	Questionnaire	Fentanyl	NA	32	NA	Death	Population-based, open cohort study	7
Dolan et al. (42)	USA	Randomized	Heroin and fentanyl	Oct 2019 and Feb 2020	124	NA	Death, suicide, and homicide	National vital statistics system	8
Schwartz et al. (43)	USA	Randomized	Methadone	24 Months	225	NA	Death	NA	7
Skolnick (44)	USA (Santa Monica, Los Angeles County)	Retrospective	Naloxone, fentanyl, morphine, and hydromorphine	24 Months	93,400	NA	Death	Medicolegal death and toxicological reports	8
Slavova et al. (45)	USA (Kentucky)	Retrospective cohort	Heroin and fentanyl	2011–2015	100,000	NA	Death	Community-based study	8
Korona-Bailey et al. (46)	USA (Tennessee)	Retrospective cohort	Fentanyl, heroin, and marijuana	March to June 2019	1,183	NA	NA	NA	6
Kinner et al. (47)	UK (British Columbia)	Retrospective cohort	Benzodiazepine and opioids for pain	2015–2017 (3 years)	6,106	NA	NA	NA	6
Abouk et al. (48)	USA	Retrospective cohort	Heroin and methadone	January 2017 to January 2019	NA	NA	NA	NA	5
Ruhm (17)	USA	Retrospective cohort	Heroin and opioid	1999–2015	NA	NA	Death	CDC and multiple causes of death	7
Quast (49)	USA (Florida)	Retrospective cohort	Amphetamines, benzodiazepines, opioids, methadone, cocaine, and heroin	2003–2017	8,633	NA	Death	MCOD data and data from the Florida Medical Examiners Commission	8
Mojica et al. (50)	USA(Atlanta, Georgia)	Retrospective cohort	Oxycodone, heroin, and fentanyl	2010–1,016	192	NA	NA	NA	6
Marshall et al. (51)	USA (California, Orange County)	Retrospective cohort	Heroin and benzodiazepine	2010–2014	1,205	NA	NA	NA	6
Stopka et al. (52)	USA (Lowell, Massachusetts)	Retrospective	Fentanyl	2008–2018	10,000	NA	Death	NA	8
Bernard et al. (53)	Norway	Retrospective cohort	Methadone	2000–2006	7,000	NA	Death and respiratory depression	NA	7
Hayashi et al. (54)	Germany (Berlin)	Retrospective cohort	Fentanyl	1998–2011	9	NA	Fentanyl-related overdose	NA	7
Gueye et al. (55)	USA (Paris and its adjacent suburbs, Texas)	Retrospective cohort	Methadone, propoxyphene, and cocaine,	1995–1999	501	NA	NA	NA	6
Bogdanowicz et al. (56)	UK (South London)	Retrospective cohort	Opioid and alcohol	NA	4,837	NA	NA	NA	6
West et al. (57)	West Coast of the USA (San Francisco, North Carolina)	Retrospective cohort	Fentanyl	2009–2019	1,510	NA	NA	NA	6
Rodieux et al. (30)	USA	Retrospective cohort	Tramadol	NA	231	NA	NA	NA	6
Van Hout (58)	Ireland	NA	Codeine	NA	156	NA	Death and psychiatric comorbidity	Maudsley case register	6
Fodeh et al. (59)	USA	NA	NA	NA	1,677	NA	Death	NA	7
Xiang et al. (14)	USA	NA	Oxycodone, hydrocodone, psychotropic agents, analgesics, antipyretics, and antirheumatics	NA	100,000	NA	Death, suicide, or accident	case	6
Nielsen et al. (60)	Denmark	NA	Methadone	January 2008 to December 2011	103	NA	NA	Pre-hospital, hospital, and postmortem data	7
Powell and Pacula (61)	USA (California)	NA	Oxycontin, oxycodone, fentanyl, and heroin	1997–2017	NA	NA	Death	NA	4
Espelt et al. (39)	Spain (Barcelona)	NA	NA	NA	NA	NA	NA	Opioid-related incidents and fatal opioid overdose data	2
Ruhm (15)	USA	NA	NA	NA	NA	NA	Death, respiratory depression, and anesthesia	Internet searches	3
Wares et al. (62)	USA (Philadelphia)	NA	NA	NA	NA	NA	NA	In-depth interviews	2
Mattson et al. (63)	USA	NA	NA	July 2016–June 2017	NA	NA	Death	NA	4
Vickers-Smith et al. (64)	Kentucky	NA	NA	NA	NA	NA	Death.	NA	3
O’Donnell et al. (65)	Canada (British Columbia)	NA	NA	NA	NA	NA	Death and fatal overdose	NA	3
Dayton et al. (5)	USA (Baltimore, Maryland)	NA	NA	NA	577	NA	NA	USP convention	5
Burke et al. (66)	USA (Massachusetts)	NA	NA	2011–2014	2,154,426	NA	NA	NA	4

NA, Not available.

**Figure 2:**
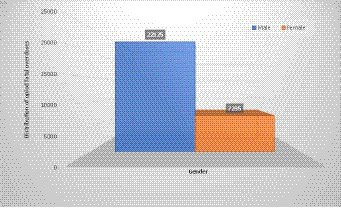
Gender distribution of multinational prevalence estimate of opioid fatal overdose.

**Figure 3:**
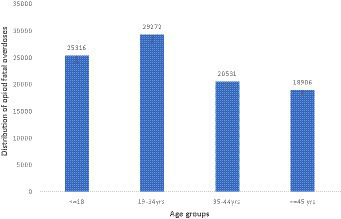
Age distribution of multinational prevalence estimate of opioid fatal overdose.

Among the 15 studies that reported on the gender of the multinational prevalence of opioid fatal overdose, we observed that male individuals have a higher prevalence than female individuals, i.e., there are 22,125 (15.79%) male individuals compared to 7,235 (5.17%) female individuals, as shown in Figure 2. On the other hand, only 14 studies reported the ages of the participant in their articles. The age distribution indicates that the 18-34-year-old age group is more at risk of opioid fatal overdose with a prevalence of 29,272 (31.13%), followed by the less than 18-year-old age group with a prevalence of 25,316 (26.92), as shown in Figure 3. However, among the selected articles, we found that a total population of 101,911 were tested for fatal opioid overdose, with a prevalence of 15,022 (14.74%).

#### Quality assessment

The quality evaluation ratings of the included studies are presented in Supplementary Table S1, displaying specific information on the evaluation questions listed as per domain for each article. However, the NOS variables’ comparability awarded 0 stars to any of the evaluated research articles because comparative studies were not included in the articles that were selected. The quality ratings for the included studies range from 6 to 8. Of the possible 9 points, ten studies received 8 points, seven received 7 points, and two received 6 points.

#### Global/multinational distribution estimate of opioid fatal overdose

**Figure 4:**
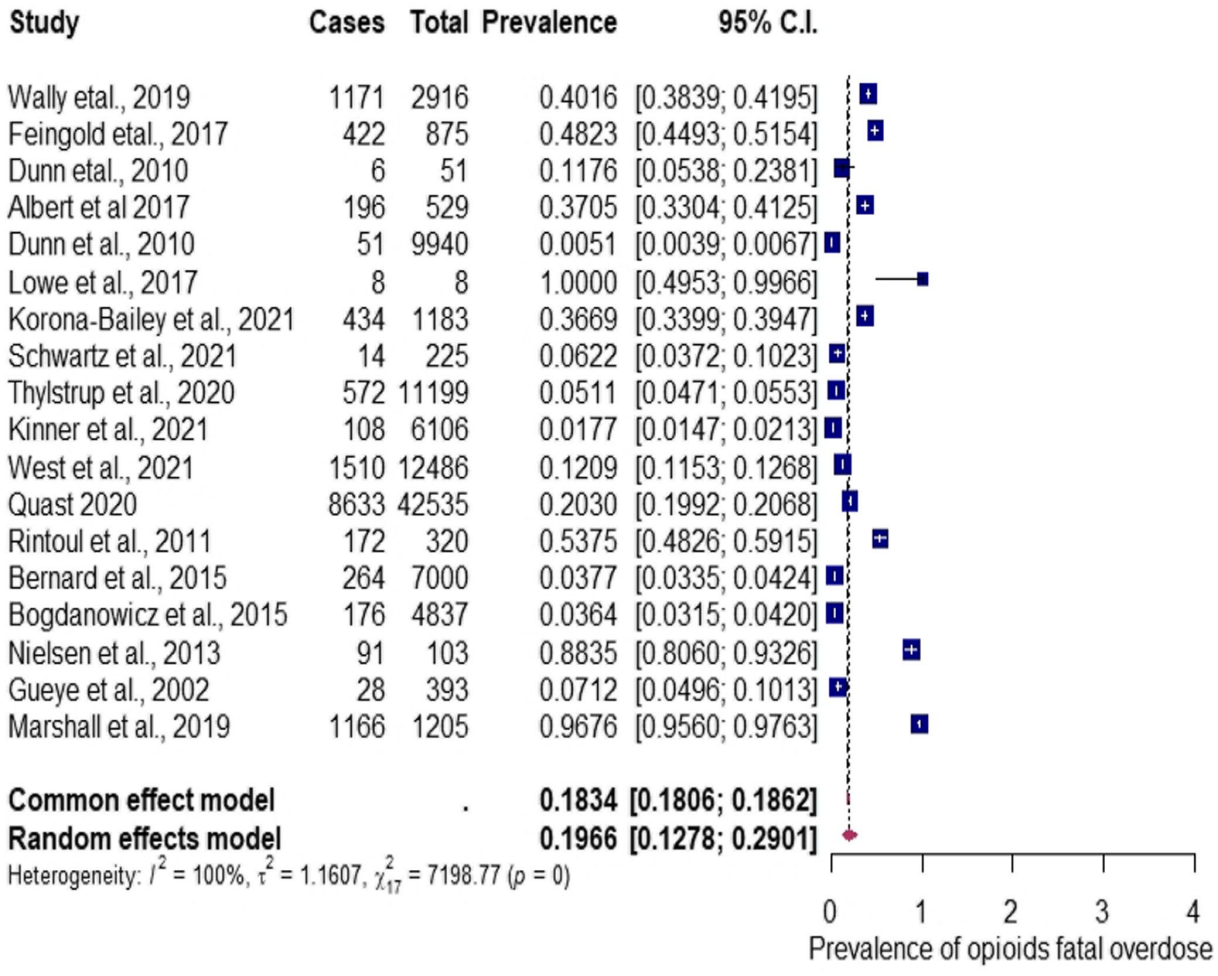
Forest plot for the prevalence of opioid fatal overdose.

**Figure 5:**
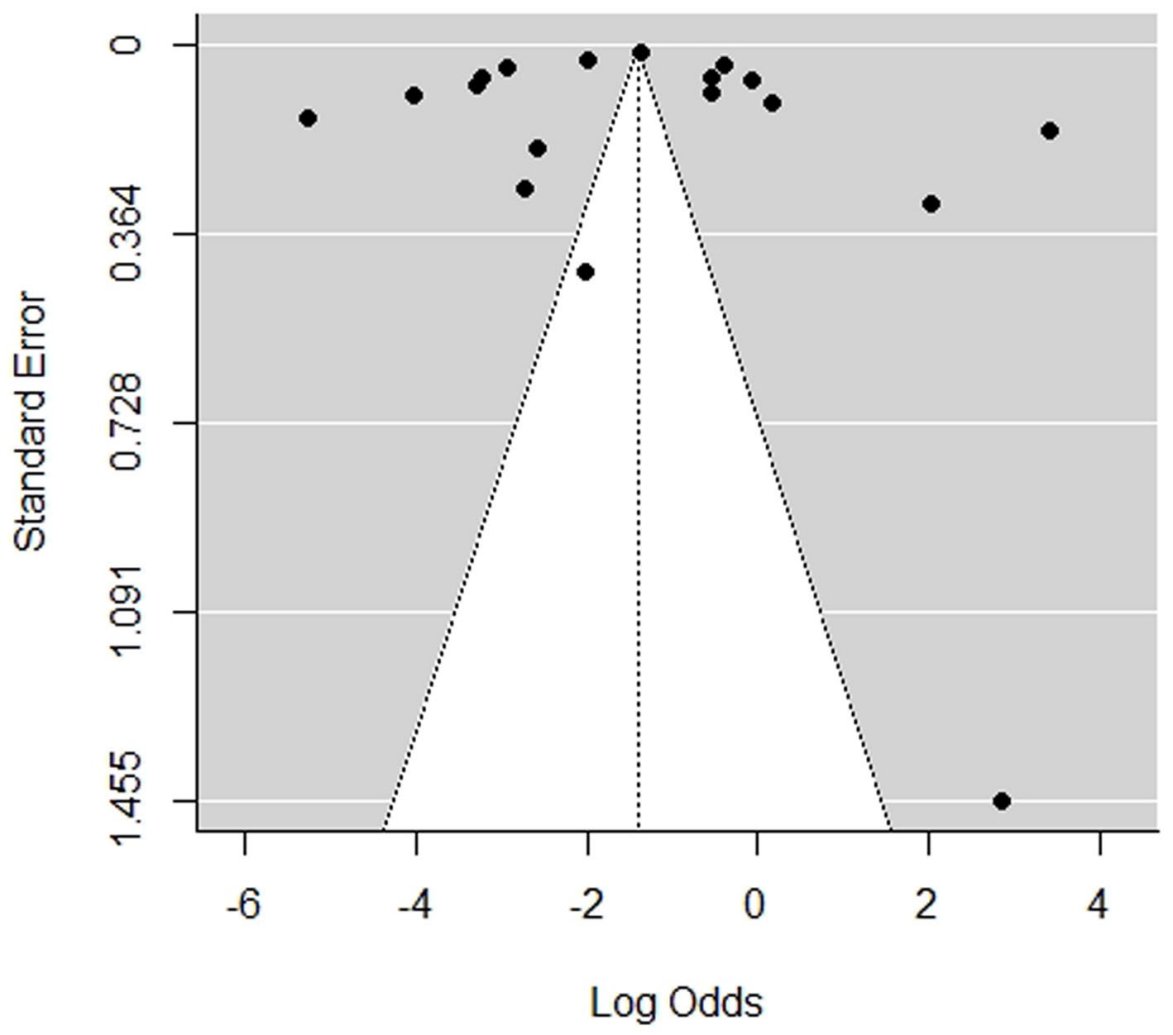
Publication bias shown in the funnel plot for the multinational prevalence of opioid fatal overdose studies.

The 18 meta-analyzed studies on the multinational prevalence of opioid fatal overdose indicate an estimate of the overall pooled proportion estimate of 19.66%, with 95% CIs (0.13–0.29), *I*^2^ = 99.76% determined using the random-effects model, and Q statistic of 7198.77 (*p* < 0.0001), which implies a high distribution of fatalities between studies and a significant difference in the effect size of the included studies. Therefore, the overall analysis has considerable heterogeneity in the meta-analysis of opioid fatal overdose prevalence, as shown in Figure 4. The Egger test model revealed that the publication bias was insubstantial (*p* = 0.015) via funnel plot asymmetry, as shown in Figure 5. The publication bias was represented using a funnel plot. In the funnel plot shown in Figure 5, each point indicates a different analysis of the specified connection. The vertical line indicates the mean effect size. The asymmetrical distribution of the scores indicates publication bias. While the funnel plot asymmetry was tested for linear regression using the Egger test model, the results showed a moderately insignificant publishing bias (*z* = 2.4273, *p* = 0.015; Figure 5). Alternatively, the rank correlation test using Kendall’s model regarding the asymmetry in the funnel plot shows that tau = 0.0065 (*p* = 1.0000).

#### Variations in the multinational prevalence/distribution of opioid fatal overdose: subgroup analysis

The subgroup analysis was used to investigate possible variations and contributions to the prevalence of opioid fatal overdose. The variables of the subgroup analysis include countries (USA, UK, Denmark), or others (namely, Israel, Spain, Canada, Australia, Norway, and France), study design (cohort or other), study period (months or above 1 year), or opioid type [one specific opioid or mixed (more than one opioid, e.g., a combination of methadone, fentanyl, heroin, etc.)]. The result of subgroup analysis determined by the categorical variable study design (cohort or others) shows others as having the highest prevalence estimate of 34.37, 95% CIs (0.1600–0.5901); *I*^2^ = 97.04%, compared to that of the cohort with an estimate of 16.74, 95% CIs (0.1020–0.2624); *I*^2^ = 99.81% (see details in ST 3). The results of the subgroup analysis determined by sample size shows the highest prevalence of 34.66, 95% CIs (0.2039–0.5234); *I*^2^ = 97.82%, for the less than 1,000 sample size compared to the more than 1,000 sample size with a prevalence of 12.28, 95% CIs (0.0675–0.2131); *I*^2^ = 99.85%. The results of the subgroup analysis determined by opioid types reveals the highest prevalence estimate of 24.39, 95% CIs (0.1459–0.3786); *I*^2^ = 99.76%, in individuals that use mixed opioids, 04.54, 95% CIs (0.0281–0.0727); *I*^2^ = 71.10%, in those that use only the methadone opioid type, and 12.09, 95% CIs (0.0125–0.5990) in those that use the fentanyl opioid type, for which no *I*^2^ table was computed since there was only a single study on fentanyl. The results of the subgroup analysis determined by study period (months or above 1 year) indicates the year to have the highest prevalence of 22.03, 95% CIs (0.1401–0.3287); *I*^2^ = 99.77%, compared to months, which has the calculated prevalence of 10.80, 95% CIs (0.0119–0.5500); *I*^2^ = 99.80%. The results of the subgroup analysis determined by countries indicates the highest prevalence ranging from 50.86, 95% CIs (0.1742–0.8355), for UK, 36.99, 95% CIs (0.1051–0.7458); *I*^2^ = 99.78%, for Denmark, 15.30, 95% CIs (0.0386–0.4483), *I*^2^ = 99.77%, for others (namely, Israel, Spain, Canada, Australia, Norway, and France), to 13.38, 95% CIs (0.0812–0.2127), *I*^2^ = 99.67% for the USA. The detailed forest plots for the subgroup analysis are shown in Supplementary Figures S2–S6.

#### Source of heterogeneity analysis for the multinational prevalence of opioid fatal overdose: meta-regression

Five covariate factors were examined to determine the likely sources of heterogeneity observed in the overall prevalence estimates of the included studies. The univariate meta-regression analyses indicate that the overall multinational prevalence of opioid fatal overdose was not significantly associated with the countries (USA, UK, Denmark, or others), study design (cohort or other), study period (months or above 1 year), opioid type (one specific or mixed). In the covariates analysis, the *p*-values and R^2^, which refers to the amount of heterogeneity accounted for, countries, study design, study period, and opioid type were *p* = 0.1420; R^2^ = 0.00%, *p* = 0.1241; R^2^ = 0.53%, *p* = 0.2311; R^2^ = 0.00%, and *p* = 0.1216; R^2^ = 0.00%. Nonetheless, the sample size (less than or greater than 1,000) (*p* = 0.0098; R^2^ = 3.83%) was significantly associated with the multinational overall prevalence estimate of opioid fatal overdose.

## Discussion

This study provides the first globally synthesized scientific evidence on the prevalence or distribution of opioid fatal overdose. The potential heterogeneity examined includes the prevalence or distribution of categorical variables, namely, countries or regions, opioid types, study design, sample size, and study period, and shows evidence of temporal demographic variables (gender and age group). We estimated the global/multinational prevalence of opioid fatal overdose to be 19.66%, with the UK having the highest distribution and an upward trend among the mainly implicated 18-34-year-old age group.

### Scientific evidence and efficient data utilization for opioid crisis prevention/management strategies

The overall findings of this study highlight that opioid fatal overdose is, at present, one of the common causes of death and a pandemic that places a burden on the public. Thus, there is a need for opioid mortality data documentation and precise and exhaustive epidemiological data worldwide. Few large-scale epidemiological studies that have been conducted on opioid fatal overdose, which, unfortunately, are concentrated on particular regions, restricted populations, and partial epidemiological variables. The dearth of such studies is often due to low public awareness, poor health policies, and a lack of associated health surveillance systems, resulting in an inadequate synthesis of the evidence and outcomes. However, evidence data from various public health and public safety sources is crucial to the success of opioid overdose prevention programs and policies, both at the regional and international levels. Over the years, specific long- and short-term goals form the part of treatment that focuses on three generalized aims shared by all specialized drug misuse treatment programs: decreasing substance abuse or creating a life devoid of substances, optimizing the functioning of several facets of life, and preventing relapses or minimizing their frequency and severity (67). The treatment models and approaches include *medical, psychological, and sociocultural* ones that utilize four major treatment approaches: *The Minnesota model of residential chemical dependency treatment*, *drug-free outpatient treatment*, *methadone maintenance -- or opioid substitution – treatment*, and *therapeutic community residential treatment* for alcoholism and drug dependence, which is also implicated in opioid addictions. The current specific opioid treatments strategies are the Enhanced Treatment-as-Usual (ETAU) program, detoxification with methadone and referral to treatment in the community (43), the use of take-home naloxone (THN), medication-assisted treatment (MAT) (5), established needle and syringe programs (NSPs) and opioid substitution therapy programs in most countries such as Mauritius, Senegal, Tanzania, Kenya, and South Africa (68, 69), and prescription drug monitoring programs (PDMPs). However, these programs are not relatively the same and are not available in all nations.

Using the systematic reviews and meta-analyses protocol, epidemiological data can be synthesized in a form that follows scientific reasoning for public health interventions; however, uncommon health disorder prevalence results are not readily available. Owing to the extremely low prevalence variables, epidemiological data must go through relative transformations before they are fit for a normal distribution, while the high heterogeneity of the observational studies may reduce their reliability if left untreated or without intensive analysis (20, 24, 70). Despite these challenges, we present the first-ever comprehensive global/multinational estimate prevalence of opioid fatal overdose and its key epidemiological features with the aim that it would serve as an important reference for opioid overdose prevention programs, policies, and future research health initiatives and other common or uncommon diseases.

### Global/multinational estimates of the prevalence of opioid fatal overdose

This study reviews scientific evidence on the global contemporary pooled prevalence of opioid fatalities. While our findings have shown a wide-range report of studies in the developed nations, especially in the USA, many middle- and low-income countries are poorly represented. The prevalence of opioid fatal overdose fatality is between 14.52 and 50.86% among the reported nations, which implies the impact of the current opioid crisis on public health by mainstream researchers (10, 61, 71, 72). It is commonly unknown that many factors influence the opioid crisis or pandemic, including regulatory authorities, government, stakeholders, economy, and poor health surveillance system. Opioids are responsible for the highest drug-related deaths worldwide, while cannabis products continue to be the most misused substances, with cannabis being the oldest known psychoactive plant type of over 10,000 years (73). Many countries have legalized cannabis due to its medicinal value (74). Among the 483 pharmacologically active compounds, including phytocannabinoids (74) and synthetic cannabinoids (SCs), over 224 compounds have been associated with several cognitive and psychomotor functions such as motor coordination, emotional processing, depression, and anxiety, as well as major causes of road accidents, which has a significant public health impact. More worrisome is the fact that the continuous introduction of NPS of new and powerful synthetic molecules into the market hinders forensic toxicologists’ ability to “keep up with the times” (75). In addition to the crises of illegal drug use is the risk of unexpected pharmacological effects from adulteration with other active compounds, which have raised concerns in recent times (73). The emerging trend of adulterating heroin, fentalogues, and synthetic cannabinoids (73), such as LSD-adulterated methamphetamine, heroin, and methamphetamine in Iran and xylazine-laced heroin seized in the USA (73, 76, 77), poses difficulties in their diagnosis and intensive health threats.

Opioid use disorder (OUD) and fatal overdoses denote a switch from traditional drug transit methods to internal opioid usage, a global problem that is becoming increasingly common in middle and low-income nations, particularly in the African continent. Owing to the increased use of African trade routes by global opioid trafficking channels, the use of opioids for non-clinical purposes has increased in the region (72, 78, 79). The African region has the highest rates of HIV and HPV infections (19, 80, 81), as well as the greatest shortages of medical professionals and clinics for addiction treatment. While self-medication is most common in situations with poor resources, controlled prescription drugs (CPDs) and overdosing are growing problems with little or no mainstream research attention. These problems could have a public health impact on the use of opioids in the Asian and African continents. Findings suggest that CPD use disorders are rapidly expanding in Africa and Asia (82, 83). However, many poor resource settings do not have a regular surveillance system and are faced with challenges of the diagnostic capacity of toxicological laboratories or technology advances or the required resources, thereby limiting the mainstream research report on opioid fatal overdose (49, 84). The differences between the included studies on the estimated prevalence of opioid fatal overdose may affect the validity and comparability of the findings. Nevertheless, the differences are explained in the following paragraphs.

One difference is the inclusion of opioid fatal overdose patients based on death outcomes and diagnostic methods. Most of the reported methods among studies include a death certificate, medical examiner’s commission, office of the chief coroner, national vital statistics system, CDC, and Maudsley case register (12, 15, 37, 42, 44, 49). While the most frequently used method is postmortem toxicology or toxicological tests (autopsy) (28, 36, 60), which may vary concerning the drugs that were detected. Furthermore, the nature and characteristics of the death-causing drugs may make interpreting the test results difficult. Additionally, problems could occur if the person spent a long time in the hospital before death or the body was not found for a long time after death. Location of death, ethnicity, and confidentiality concerns all influenced data availability, which affected the overall prevalence observed. However, our study provides scientific evidence of the global/multinational prevalence estimate of opioid deaths, and further examining any of the reported discrepancies in this category of fatal overdoses could provide crucial insights for management strategies. The age at diagnosis of the patients’ fatal overdose was found to be high in the less than 18-year-old and the 18-34-year-old age groups, which is similar to the current report on other studies (15, 44). In a previous study, the narrative involved patients between the ages of 40 and 50 in the years 1999–2013 (85–88), and this increase is age can be attributed to a wide range of economic and social causes (85). Other studies have suggested that increasing income inequality, global commerce, stagnant wages, rising unemployment, and economic and social degradation are important in increasing drug use (89, 90). Moreover, other substantial bodies of literature suggest that economic conditions influenced the abuse of drugs (16, 91–93). The increase in drug use observed in the <18-year-old age group may be due to influence or pressure from peers and friends, curiosity, fun, family problems, relief from psychological stress, pain avoidance, pleasure-seeking, craving, habits, and impulsivity (94–97). However, people of younger ages are significantly affected by substance or opioid use compared to older ones because initiation of drug abuse at an early age is associated with high risks of acquiring dependency and other difficulties during adult life. In addition, our findings reveal an increased use among male individuals compared to female individuals (15). However, the reason for the increase in use by male individuals is not clear to us. Prescription and illicit opioid use have played equal roles for the increase in fatal overdoses in older female individuals. In this study, age is emphasized as, unsurprisingly, opioid overdose is among the leading causes of death among people aged less than 18 years and those aged more than 45 years and is a crucial contributing factor to the reduction of the average lifetime (44, 98, 99). Second, the included studies are too diverse in study designs and sample size for a comparison between case report studies and population-based observational, large-scale national and prospective or retrospective cohort studies.

The differences in study design may be due to the considerable gap in medical care and therefore data availability, cause of death documentation, and medical health recording systems. Most affluent nations have comprehensive referral database structures and medical documentation systems, making it possible to conduct excellent prevalence studies. On the other hand, many developing countries are restricted to regional or hospital-based studies and lack regular surveillance systems (19, 24). Some of us may speculate if these restrictions could explain why, despite the African trade routes being used as global opioid trafficking channels, which may have impacted the region’s non-clinical uses (72, 78, 79), no characteristic epidemiological studies on fatal overdoses are reported in the region. Therefore, middle- and low-income nations need enhancements of screening methods for reporting opioid/drug fatal overdoses and the creation of public health initiatives or training in overdose prevention programs.

Third, regarding opioid types, our study showed various types of opioids with significant clinical implications. The most common type of opioid in toxicological testing was fentanyl, while the most prevalent types were mixed types of two or more and/or alcohol, including fentanyl, heroin, oxycodone, isotonitazene, and methadone. Research has shown that heroin and illicit opioids are responsible for the majority of drug-related fatalities, with overdose ranking as the top killer of opioid users (39). It has been shown that the extensive increase in the number of deaths is related to the use of synthetic opioids (such as oxycodone, fentanyl, pethidine, methadone, and tramadol), which is well established in developed nations such as the USA and Canada, as the misuse of prescription drugs are of emerging global concern (38, 100). In addition, increases in the illicit use of remedy opioids have been characterized as a paradigm shift from heroin by way of street opioid abuse in high-income nations such as the USA, Canada, and Australia (101). The significant increase in the usage of OxyContin and other prescription opioids has been largely censured for the spike in drug-related fatalities (102, 103). However, several variables could contribute to an increase in the number of deaths from illicit opioids in developed nations, including the switch to heroin, the release of an abuse-deterrent formulation by OxyContin in 2010, the low street price of heroin, and the increasing distribution of Mexican heroin into rural and suburban regions, and the assortment of heroin and illicit fentanyl has greatly increased the danger of fatalities (104–106). On the other hand, prescribing patterns of opioid use for chronic pain management also contributed to the epidemic in 2000, which was further escalated with the supply of potent synthetic opioids in 2013 (32, 107, 108). Moreover, the concomitant use of other CNS stimulants or polydrugs significantly increases opioid fatal overdoses, yet the effect of illicit opioid misuse is underestimated. Other epidemiological characteristics such as race or ethnicity, marital status, education, employment income, or economic status could not be determined, due to insufficient data availability from the primary studies (109).

### Opioid misuse-related fatalities and public health implications

The opioid epidemic has a complicated effect on public health that is intertwined with a variety of variables, including social determinants of health and mental health, increasing deaths of overdose among teenagers, childhood trauma (9, 110), and transmission of blood-born infections due to sharing of heroin injection equipment (111). There are many societal components of the public health impact of opioid use disorder, including loneliness, a higher risk of suicide, and, at the neighborhood level, less social capital. Neonate toxicity, such as the risk of neonatal abstinence syndrome (NAS) or opioid prenatal exposure, may result in poor fetal growth, preterm birth, or stillbirth. The study of Jilani et al. (112) reported a 433% increase in the incidence of NAS between 2004 and 2014.

**Figure 6:**
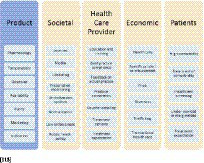
Opioid public health implications and multifactorial contributing factors.

Nevertheless, the public health problem with opioids is multifaceted. There are many various pillars of factors ranging from product type, societal impact, health care provider, and economic to patients, and each of these factors contributes a different constituent to the opioid global crisis (109) (Figure 6). However, this study further advocates for a comprehensive public health approach that articulates the multidimensional opioid crisis to offer an integrated approach to prevention and management.

The global public health opioid crisis requires considerably more financing and leadership. While organizations/societies such as the College on Problems of Drug Dependence (CPDD), the American Society for Clinical Pharmacology and Therapeutics (ASPET), the American College of Clinical Pharmacology (ACCP), and the American Society for Pharmacology and Therapeutics (ASPET) may provide scientific leadership and focus when addressing a public health issue associated with opioid fatality (109). A national scientific summit might influence the direction of future research.

### Implications of opioid misuse and illicit trafficking on the economy and society

The connections between illegal drug trafficking enterprises and terrorist or insurgent groups—whether for financing operations, garnering political support, or subverting an established government—are a topic of significant debate (113). Evidence suggests that many terrorist and rebel groups traffic illegal substances primarily for financial gain or pragmatic reasons. Even while they may have ideological objections to the drug trade itself, several people, especially in the South American countries where coca is grown, use the money they make from the cocaine trade to further their political influence and to fund their operations. For example, the Colombian government estimates that between one-third and one-half of the activities of the leading guerilla group in the nation, the Fuerzas Armadas Revolucionarias de Colombia (FARC) (Colombian Revolutionary Armed Forces), are funded by narcotics drug trafficking (114, 115). Several groups in Central America, Afghanistan, Myanmar, Sri Lanka, and Thailand are reported to have comparable goals and make a sizable profit from human trafficking (114, 115). Unlawful drug proceeds, whether or not they are laundered, have the potential to enter the formal economy and then the political system, jeopardizing the establishment and smooth operation of civil society and resulting in chaos and societal collapse (114, 115). In some producer/trafficking nations, reports have shown that drug money infiltrates the “last fissures of the political system, society, the economy, along with cultural and sports activities… to obtain public support and respect, and, of course, providing an ideal vehicle for money-laundering” (114, 115). More unsettling is that the amount of money under criminal control puts governments at particular risk, especially in developing nations with insufficient domestic capital and security markets to handle such sums without rapidly becoming reliant on them. In South Asia, where tramadol is mainly produced, there are indications that transnational organized criminal networks move it across the Gulf of Guinea to parts of the Sahel that are primarily under the control of terrorist and armed groups (116). The Nigerian law enforcement officers and UN officials also reported a connection between tramadol misuse and the Boko Haram terrorism group, which contributed to the destabilizing and violence in northeastern Nigeria in 2017 (117, 118).

Research has shown a substantial correlation between substance misuse and food insecurity, with up to 70% of those with abuse or addiction to drugs being found to be food insecure (119). Food insecurity was linked to noticeably higher chances of having used the majority of different drugs (120). Heroin and other opioids frequently cause sugar cravings, which lead to unhealthy eating habits and an increase in body mass index (BMI) in drug users (119). However, food insecurity resulting from drug abuse and mental disorder patterns has profound consequences that impact not just addicts but also their families, especially children, who may experience life-altering consequences (119). It is relatively not unusual for people who struggle with alcoholism or drug addiction to skip meals, sometimes even for entire days, in order to use the money to feed their addiction or drugs.

### National and international approaches and strategies in the detection and prevention of opioid misuse

The findings in this study highlighted the poor screening/detection methods for reporting opioid misuse to fatal overdoses, and the most important method is the detection of shipping routes by global opioid trafficking channels. The United Nations Office on Drugs and Crime (UNODC) and European Monitoring Centre for Drugs and Drug Addiction (EMCDDA) have reported the worldwide spread of heroin, LSD, MDMA (“ecstasy”), and methamphetamine and growing emergence of amphetamine-type stimulants (ATS), other synthetic opioids from morphine, butorphanol, and three principal classes of drugs: piperidine derivatives (containing fentanyl); aralkylamine derivatives (including tramadol); cyclohexane derivatives (e.g., AH-7921 and U-47700), and 62 novel psychoactive substances (NPS) with opioid effects, including 48 fentanyl analogs, in the past 10 years (121). Given the rapid growth and structural diversity of synthetic opioids in the global medication market, it is imperative to implement international control measures aimed at the most hazardous and common misuse as top priority. The WHO has proposed a surveillance system concept that would work with different bodies, such as UNODC and EMCDDA, to share data collaboratively, increasing the number of compounds reviewed throughout the prioritization phase (121). Building national capacity for data collection and management will help ensure that EU member states can collect and handle data on synthetic substances, opioids, and NPS. The Early Warning System of the EMCDDA is relevant and dramatically contributes to collecting spectral data (infrared, GC/MS, and nuclear magnetic resonance imagings) on NPS substances and opioid derivatives from around the globe (121). The various European forensic institutes and laboratories operating for customs enforcement provide most of the data and information on NPS. International databases of NPS have also been established and predominantly elusive for the international control system across international borders after being scrutinized by the WHO Expert Committee on Drug Dependence (ECDD) in 2014 (World Health Organization, 2014), and 40 NPS had been scheduled by the International Drug Control Conventions in 2018. The global SMART program is another international initiative that can help comprehensively grasp the worldwide dynamic of synthetic drug problems. To lower the dangers to public health, countries prepare and anticipate the threat posed by synthetic opioids by increasing awareness and disseminating information. Global monitoring systems may also be essential due to the problem’s complexity and international nature and the technical know-how needed for data processing and interpretation. In addition, the process of reacting to recently discovered substances or newly synthesis opioids are usually relatively slow; the Commission on Narcotic Drugs makes decisions on the substances only made once a year, which may not be sufficient to keep up with the number of new NPS/opioids that hit the market.

The European Union’s Court of Justice ruled in 2014 that substances cannot be classified as medical products unless they positively impact human health (122). Therefore, the national legislatures have implemented particular legal solutions to the NPS issue, including newly synthetic opioids, either by creating new legislation or by building on pre-existing laws centered on consumer, health, or pharmaceutical protection. The majority of EU member states’ evolving legislative initiatives to address the surge in NPS and other substance misuse are outlined in a 2018 Eurojust report (123). However, most nations lack significant operational expertise in prosecuting NPS and (pre) precursor crimes. Sometimes, the only way to regulate NPS is through administrative legislation, or it can serve as an extra regulatory foundation to fill in the gaps left by introducing new chemicals. The Eurojust report also emphasizes the importance of international agreements and customs. These two areas ought to be fully integrated into national laws. The emergence of a highly sophisticated worldwide criminal network, international drug trade routes, and the growing complexity of money laundering offences are the interrelated developments influenced by technology, and the globalization of commerce has made drug-related issues even harder for customs officials to handle situations with merely unilateral and unisectoral action. Therefore, it is necessary to evaluate the relative costs and advantages of various drug control strategies in a cross-national term, while learning and discovery have even more justification for expansion in research priorities in each nation in finding lasting solutions to be incorporated into the development of drug policies.

### Research limitations and strengths

One of the limitations of this study is that we were only able to identify fatal overdoses through the reported research protocols; however, we might have missed some ICD-10 codes, possible inaccuracies in the reporting of underlying causes of death, and particular categories of drug involvement noted on death certificates in the included studies. In addition, there was a lack of consistent or precise information as several studies were not included in the prevalence estimates. The reason for information shortfall was that they did not provide the total population, cases, expected outcome (death), specific age and sex, or study period, and almost all the studies have no information on race or ethnicity, marital status, education, employment income, or economic status. Moreover, only English-language studies were included in our analysis. As a result, the opioid fatal overdose prevalence estimated in this study may be conservative.

Our study on the epidemiological distribution of opioid fatal overdose is the first of its kind global/ multinational survey based on scientific evidence. The epidemiological survey synthesis incorporates information from various data sources, including postmortem reports, autopsy reports, toxicological data, national database, mortality registry records, WHO pharmacovigilance database, medicolegal deaths, CDC, multiple causes of death, medical examiners commission, and death certificates, to draw attention for research on an important public health burden for further studies. The information collated is comprehensive and can be utilized to learn more about fatal opioid overdoses than is known from death certificates. This study lays the groundwork for future epidemiologic studies utilizing evidence data, provides insight into preventative tactics, and highlights the necessity for more detailed data to understand opioid overdose fatalities comprehensively. It will bring regional and international prevention strategies to policymakers’ awareness. Future research may examine and compare various polydrug overdoses to opioid trends as well as evaluate the impact of non-fatal overdoses, comorbidities, and vulnerability indices to uncover the causes of opioid fatalities and prevent overdose fatalities among the minority population through awareness.

## Conclusion

Our findings suggest improving efforts to report mortality caused by specific drugs, thereby enhancing the epidemiological characteristics for policymakers and prevention strategies. In middle- and low-income nations, there should be rapid promotion and establishment of opioid overdose fatality or drug surveillance systems while also funding for comprehensive toxicology testing, training of local medical examiners or officials, and implementation systems to provide accurate overdose mortality data and identify the specific drugs of public health implications in a timely manner.

## Data availability statement

The original contributions presented in the study are included in the article/Supplementary material, further inquiries can be directed to the corresponding author.

## Author contributions

HO: Conceptualization, Data curation, Formal analysis, Investigation, Methodology, Resources, Software, Validation, Visualization, Writing – original draft. FO: Conceptualization, Funding acquisition, Project administration, Resources, Supervision, Writing – review & editing.

## Footnotes

^1^ http://www.ohri.ca/programs/clinicalepidemiology/oxford.asp
