# Supplementary material for: Multinational appraisal of the epidemiological distribution of opioid fatalities: a systematic review and meta-analysis
Source: Front Psychiatry. 2024 Jan 5;14:1290461. doi: 10.3389/fpsyt.2023.1290461 (PMC10796457; doi:10.3389/fpsyt.2023.1290461)
Supplement: Supplementary file 1 [file Table_1.DOCX]

**Multinational appraisal of the epidemiological distribution of** **opioid fatalities: a systematic review and meta-analysis**

**Hope ONOHUEAN ^1, 2 *^, Frasia OOSTHUIZEN ^1^**

^1^ Discipline of Pharmaceutical Sciences, School of Health Sciences, Westville Campus, University of KwaZulu-Natal, Durban, South Africa, OOSTHUIZENF@ukzn.ac.za

^2^ Biopharmaceutics Unit, Department of Pharmacology and Toxicology, Kampala International University Western Campus, Ishaka-Bushenyi, Uganda, onohuaen@gmail.com

**^*^** Corresponding Author: Hope Onohuean, Biopharmaceutics Unit, Department of Pharmacology and Toxicology, Kampala International University Western Campus, Uganda.

Emails: onohuean@gmail.com

**Authors Contributions**

HO and FO conceived and designed the research; HO and FO conducted the article search, extract and conduct data analysis. HO wrote the manuscript. FO revised the manuscript. All authors read and approved the manuscript.

**Acknowledgments**

The authors acknowledge Charles Omare, and Abraham Olutumininu Akiyode for their support and assistance during the data extraction and mining. We sincerely thank Dr. Kgothatso E Machaba (Scientific Writer, College of Health Science, University of KwaZulu-Natal) for assisting in editing this manuscript.

Good afternoon, ma.

This is Dr. Hope Onohuean, from KIU-WC. I was the Man that approach you at the Butabika hospital concerning a research I want to do with my students in the hospital.

Ma, I need your help, as don’t know is delaying the approval, as they requested for the full proposal and ethics approval from university, about three weeks ago, and I have provided them. But up till date, I m yet to get a positive respond.

The secretary to Deputy E.D, told me it was approved before, later he told me to send my students for follow-up.

Please ma, I m requesting you to use your office to assist me.

Thank you.
